# Topical Treatment of Second-Degree Burn Wounds with *Lactobacillus plantarum* Supernatant: Phase I Trial

**DOI:** 10.30699/ijp.2022.551202.2863

**Published:** 2022-09-04

**Authors:** Somayeh Soleymanzadeh Moghadam, Mahnoush Momeni, Samaneh Mazar Atabaki, Tahereh Mousavi Shabestari, Mina Boustanshenas, Mastaneh Afshar, Maryam Roham

**Affiliations:** 1 *Antimicrobial Resistance Research Center, Institute of Immunology and Infectious Diseases, Iran University of Medical Sciences, Tehran, Iran*; 2 *Burn Research Center, Iran University of Medical Sciences, Tehran, Iran*; 3 *Department of Biomedicine, Aarhus University, Aarhus, Denmark*

**Keywords:** Bacterial Infections, Burns, Drug resistance, *Lactobacillus plantarum*, Wounds and injuries

## Abstract

**Background & Objective:**

A burn wound is sterile immediately after injury, but opportunistic bacteria colonize the wound within 48 to 72 hours after the burn, causing delayed or failed burn wound healing. In addition, the presence of multidrug-resistant (MDR) pathogens doubles the treatment problems.* Lactobacillus plantarum *(*L. plantarum*) is a well-known antibacterial and healing agent that could be used topically to treat burn wounds.

**Case Series Presentation:**

This clinical trial study (Case Series) was performed on 20 patients with deep second-degree burns. Patients had bilateral wounds; the wound on one side of the body was considered as control (treated with silver sulfadiazine) and the other side of the body as treatment (treated with bacteria-free supernatants (BFS) of *L. plantarum*). The wounds were evaluated by microbial assessments and assessments related to healing. *Pseudomonas aeruginosa*, *Klebsiella pneumonia*, and *Staphylococcus aureus* were isolated from 4 (22.2%), 0%, and 2 (11.1%) of wounds treated with *L. plantarum* on the fifth day of the treatment, respectively. Furthermore, 12 (66.7%) of wounds treated with *L. plantarum *were free from bacteria. The need for skin grafting was the same in both treatment and control groups, but graft rejection in the group treated with *L. plantarum* was (0%) (*P*=0.02).

**Conclusion:**

Regarding eliminating or reducing infection and wound healing, bacteria-free supernatants of *L. plantarum* can be considered a possible topical treatment option in the case of second-degree burn wounds.

## Introduction

Burn injuries are a major global health problem and a significant cause of mortality and morbidity worldwide (1, 2). Burn treatment imposes great costs on patients, families, and the healthcare system. In addition, one of the most important factors that could increase the risk of nosocomial infections in burn patients is the length of hospitalization. In many cases, people with burned skin may die from bacterial infections before the burn wounds heal. Therefore, the speed of wound healing in burns is very important (3, 4).

One of the main reasons for slow wound healing and even graft rejection after burns is the colonization and growth of opportunistic bacteria on the burned skin, especially *Staphylococcus aureus*, *Pseudomonas aeruginosa*, and *Acinetobacter baumannii* (5, 6). 

These microorganisms have the ability to produce biofilms, a complex community embedded in a polysaccharide matrix. Biofilm-producing bacteria are extremely resistant to antibiotics and the host immune response (7, 8). Due to the prevalence of multidrug-resistant pathogens (MDR), treating infections with conventional antimicrobial drugs has become a global health problem associated with high mortality rates (9).

Since the 1960s, topical silver-containing antimicrobial agents have reduced morbidity and mortality worldwide, despite side effects associated with silver deposition on tissues (7, 10). Employing effective and safe methods to accelerate the wound healing process can reduce financial, physical, and psychological costs imposed on patients and their relatives (11). Currently, bacteriotherapy – the use of beneficial and safe bacteria with or without their supernatant against pathogenic organisms – is one of the new alternative methods that could be used to prevent infection, regulate inflammation, and possibly repair wounds during trauma or burns (7, 12, 13). 

Probiotics are live and beneficial microorganisms with antibacterial properties. The presence of probiotics in the body could play a preventive and therapeutic role in improving and maintaining the health of the gastrointestinal tract, reducing the use of antibiotics, and treating skin diseases, especially infectious wounds (7, 12). Using probiotics on the wound could help heal the host wound in a short time (12, 14, 15).

The exact mechanism of action of probiotics and their products is not well understood. However, their beneficial effects are manifested by several mechanisms, the most important of which are non-immunological (antimicrobial effects, competition for binding to target cells, helping enhance the mucosal barrier) and immunological mechanisms (14, 16, 17). 

One of the most important antimicrobial agents is the bacteria-free supernatant (BFS) of probiotics, which induces apoptosis in cells. The inhibitory effect of BFS is directly related to its concentration. Also, independent of acidity due to the presence of a higher number of metabolites produced in the supernatant (18, 19).

Today, among all probiotic species, *Lactobacillus spp.* and their supernatants are used in most probiotic research. According to the results of various studies, bacteriotherapy with lactobacilli could be considered a suitable alternative to other drugs and used in the treatment or prevention of some diseases and infections, especially traumatic wound infections in animal models; it could be used as an important medical strategy (2, 12, 20).

Some Lactobacillus strains and their products exhibit significant inhibitory activities against multidrug-resistant (MDR) bacteria (2).

*Lactobacillus plantarum* (*L. plantarum*), is a widespread bacterial species. *L. plantarum 299v* secretes antibacterial substances such as lactic acid, benzoic acid, hydrogen peroxide, bacteriocins, and short-chain fatty acids (SCFAs) (2, 21). *L. plantarum* lowers the intestinal pH and creates an unfavorable environment for the growth of pathogens (1, 2). BFS of probiotics contains antibacterial agents that regulate the population composition of normal flora and thus reduce the incidence of bacterial infections. The use of probiotic products could be considered an important medical strategy due to their bactericidal effect (5, 22).

The role of acetic acid and hydrogen peroxide in the BFS of probiotics has been proven in controlling infection and healing burn wounds, contributing to the healing process of burn wounds in a short period of treatment. Therefore, BFS of probiotics could be used as a topical alternative to antibiotics (5, 18, 19).

Due to the need for effective and safe topical treatments for burn wounds, the current case series study was conducted to treat second-degree burn wounds using *L. plantarum* cell-free supernatant.

## Clinical Trial Methods

T The current clinical trial study was performed on 20 male and female burned patients who were referred to Motahari burn hospital, Iran, from October 2019 to February 2020. Patients were in the age range of 18 to 50 years. Two of the 20 patients left the study with their consent, and the project continued with 18 patients. The selected patients were with deep second-degree burns, and the percentage of the total body surface area (%TBSA) of burns was between 10-20%. All patients were informed about the study's aim, and they entered the study with their consent. The study was approved by Iran University of Medical Sciences Ethics Committee with code IR.IUMS.REC.1397.614.

**Inclusion Criteria**

- Patients with deep second-degree burn

-Patients with %TBSA between 10-20%

- Patients between 18 to 50 years-old

-Informed consent: if any of the patients did not want to continue the treatment with the method presented in the current study, they were referred to the physician for treatment with standard methods.

**Exclusion Criteria**

-Patients with primary infection

-Patients with underlying diseases, including diabetes, high blood pressure, kidney disease, and a history of seizures

-Taking oral antibiotics during treatment; if the wound became infected during treatment, or the person had to take antibiotics for any other reason

 Patient's request not to continue the treatment with the method presented in the study and to withdraw from the study

**Study Design and Patients**

A total of 18 patients were selected with bilateral burns (two feet or two hands), of which one hand or foot (left) was assigned to the control group, and the other hand or foot (right) was assigned to the experimental group. The control group (received routine treatment) and the experimental group (received the probiotic product). The study groups were as follows: 

- Control group: treated with the common hospital treatment method (wound washing with normal saline + silver sulfadiazine ointment + dressing) for 7 to 10 days.

- Treatment group: treated with the method presented in the study (wound washing with normal saline + ointment containing *L. plantarum* product + dressing) for 7 to 10 days.

**Collecting Demographic and Burn Information of Patients**

The patient's demographic information, including gender and age, was collected. Also, other data related to patients, including admission date, TBSA percentage, and burn degree, were checked, and recorded (Tables 1 and 2).

**Preparation and Cultivation of Probiotic Bacteria**

In the current study, *L. plantarum 299v* (DSM9843) strain was obtained from the Iranian Biological Resources Center, Iran. Lactobacillus strains were grown anaerobically on De Man, Rogosa, and Sharpe (MRS) broth and agar at 37°C for 48 h and maintained at -20°C (23). 

**Bacteria-free Supernatant Preparation**

To prepare BFS probiotic, *L. plantarum 299v* strain was cultivated in MRS broth medium (MERCK, Germany) for 48 h under anaerobic conditions. The supernatant was prepared by centrifuging the probiotic suspension at 3000 rpm for 20 min in a centrifuge (Sigma 3-16k, Germany). Then, the centrifuged suspension was filtered through a 0.22 μm pore size sterile filter. The BFS of the probiotic was prepared and used freshly (24).

**Preparation of Ointment Containing BFS**

After centrifugation of the probiotic supernatant and filtration, the resulting solution was added to Eucerin (Eucerin®, Original Healing Cream, Germany), kept at 4°C, and used for topical treatment of patients.

**Treatment and Wound Healing**

All patients were treated using Eucerin + BFS and silver sulfadiazine ointment on the right foot or hand right hand as well as left foot or hand, respectively. The whole burn surface was covered with Eucerin + BFS or silver sulfadiazine ointment. Patients were treated topically once a day for 7 to 10 days. Treatment of patients continued until skin grafting. However, some patients were treated entirely without the need for skin grafting. During the treatment process, patients were visited by infectious disease physicians as well as surgeons. Clinical signs, as well as the morphology of their wounds, were examined. If the patient´s condition or wound worsened, the current treatment was changed to routine treatment.

To evaluate the wound healing process, the following criteria were considered: reduction of bacteria (clinical signs as well as wound morphology), reduction of exudate levels, reduction of pain and odor, the advent of granulation tissue, as well as epithelialization (25). On the fifth day of treatment, a wound culture test was performed for the patients' wounds with a sterile swab (3). Then, the bacteria isolated from the wounds were identified, and their microbial resistance patterns were investigated. Antibiotic sensitivity patterns of different isolates were investigated by disk diffusion (dd) method using Mueller Hinton agar medium (MHA, Merck, Germany) and antibiotic disks (Mast Group Ltd., Merseyside, UK) based on the recommendations of the Clinical and Laboratory Standards Institute (CLSI, 2017) (2). In the current study, the antibiotic disks used included Vancomycin (30 μg), Ciprofloxacin (30 µg), Gentamicin (10 µg), Amikacin (30 µg), Imipenem (10 µg), Ceftazidime (30 µg), Clindamycin (2 µg) Cefalexin (30 µg), and Erythromycin (15 µg).

**Statistical Analysis**

The data were analyzed by SPSS software, Version 20.0 (SPSS Inc., Chicago, IL., USA), and Excel 2013. Comparisons between groups were performed using non-parametric statistical tests, including the Chi-square test. A P-value of < 0.05 was considered statistically significant.

## Results

The current study evaluated 18 male and female patients with deep second-degree burn wounds and %TBSA between 10-20%. All patients were in the age range of 18 to 50 years (Tables 1 and 2). In this study, 16 (88.88%) patients were male, and 2 (11.11%) were female. In addition, 6 (33.33%) and 12 (66.66 %) of the selected patients were 18 - 30 and 31 - 50 years, respectively. The percentage of TBSA was 10% in 7 (38.88%) of patients and 11-20% in 11 (61.22%) of patients.

**Fig. 1:**
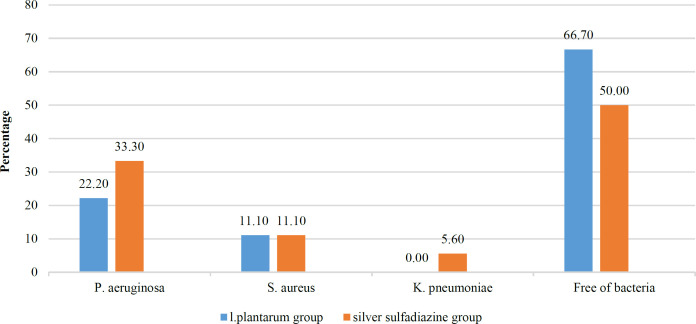
The percentage of bacteria isolated from the patients' wounds in both treatment and control groups on the fifth day of treatment. The difference was observed among control and treatment groups in terms of the presence of bacteria in the wound (phenotypic assay)

As shown in Figure 1*, K. pneumoniae* strains were not detected in the group treated with* L. plantarum* on the fifth day of treatment. *P. aeruginosa* was isolated from 4 (22.2%) wounds treated with *L. plantarum*. At the same time, this percentage was 6 (33.3%) in the silver sulfadiazine receiving group. The percentage of wounds colonized by *S. aureus* was the same in treatment and control groups 2 (11.1%). Also, 12 (66.7%) of wounds treated with *L. plantarum* were free from bacteria on the fifth day of treatment. This percentage was 50% in the other group; however, there was no significant difference in this regard between the two groups based on the Chi-square test results (*P*= 0.49) (Figure 1). Then, the antibiotic resistance pattern of a different strain isolated from the patient's wounds in both groups was evaluated on the fifth day of treatment (Table 1). According to the results shown in Table 1, 100% of *P. aeruginosa* strains isolated from both treatment and control groups were resistant to antibiotic discs, including CIP, AK, CAZ and IMI. The percentage of resistance to GM was lower in *P. aeruginosa *strains isolated from the* L. plantarum* treated group compared to those isolated from the silver sulfadiazine treated group ((9 (50%) versus 12 (66.7%)). The percentage of antibiotic resistance of *S. aureus* strains to antibiotic discs, including VAN, CIP, AK, CLI, ERY, and CN, was 0% in both treatment and control groups. *K. pneumonia* strains were not detected in the group treated with *L. plantarum*, while 100% of *K. pneumonia* strains isolated from the control group were resistant to CIP, GM, and CAZ.

Antibiotic resistance among *P. aeruginosa*, *S. aureus,* and *K. pneumonia* strains isolated from the wounds in both treatment and control groups (by disk diffusion test). VAN: Vancomycin, CIP: Ciprofloxacin, GM: Gentamicin, AK: Amikacin, CAZ: Ceftazidime, CLI: Clindamycin, ERY: Erythromycin, IMI: Imipenem, CN: Cephalexin.

In the next step, patients whose wound cultures were negative and required skin grafting were candidates for skin graft surgery. After treatment or skin grafting and discharge from the hospital, patients were followed up for two weeks. Overall, the results showed that 10 (55.6%) patients needed skin grafting, and the need for skin grafting was the same in both treatment and control groups. Therefore, no significant difference was observed between the two groups (Chi-square test). In addition, the rate of graft rejection in the group treated with silver sulfadiazine was 4 cases (22.2%). In comparison, this rate in the group treated with *L. plantarum* was 0%. This difference was significant between the two treatment and control groups (*P*=0.02) (Table 2).

**Table 1:** Evaluation of antibiotic resistance of different strains isolated from the wounds of patients on the fifth day of treatment

Strain	Percentage of resistance (%)									Groups
	VAN	CIP	GM	AK	CAZ	CLI	ERY	IMI	CN	
*P. aeruginosa*	-	100	50	100	100	-	-	100	-	*L. plantarum*
	-	100	66.7	100	100	-	-	100	-	Silver sulfadiazine
*S. aureus*	0	0	-	0	-	0	0	-	0	*L. plantarum*
	0	0	-	0	-	0	0	-	0	Silver sulfadiazine
*K. pneumoniae*	-	-	-	-	-	-	-	-	-	*L. plantarum*
		100	100	0	100	-	-	0	-	silver sulfadiazine

**Table 2:** A descriptive evaluation of skin grafting and graft rejection

Group		Doing skin grafts^ a^ N (%)		Total		Rejection of skin grafts ^b^ N (%)		Total
		Yes	No			Yes	No	
*L. plantarum*	10 (55.6)		8 (44.4)	18 (100)		0 (0)	18 (100)	18 (100)
silver sulfadiazine	10 (55.6)		8 (44.4)	18 (100)		4 (22.2) *	14 (77.7) **	18 (100)

*and ** were calculated for patients who received skin grafts.

a: Chi-square: Value: 0, : 1, Sig.: 1

b: Chi-square: Value: 5, : 2, Sig.: 0.02

## Case Presentation

**Case 2**

**Fig. 2:**
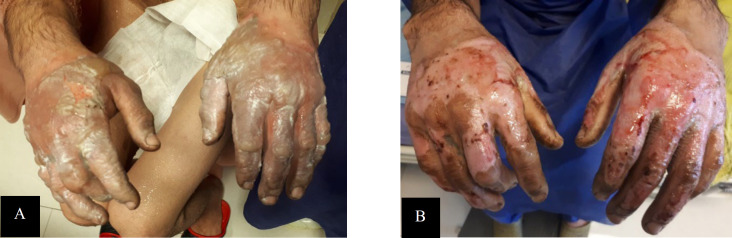
**Case 2: **Treatment of the right hand with *L. plantarum *supernatant and the left hand with silver sulfadiazine: A) after 48 h and B) after 7 days of treatment

The patient was a 40-year-old man with deep second-degree burn wounds on both hands, including the surface of the hands, wrists, and fingers. The percentage of TBSA was 10% in the patient; also, extensive fluid-filled blisters appeared on the surface of his hands within 48 hrs. after the burn. The patient received daily ointment containing *L. plantarum* supernatant on his right hand and silver sulfadiazine ointment on his left hand for 7 days. The wound culture test performed before hospital discharge was negative for both hands. The wounds on both hands healed on the tenth day of treatment without needing skin grafting (Figure 2). The patient continued the treatment until complete recovery, even after discharge.

**Case 17**

**Fig. 3:**
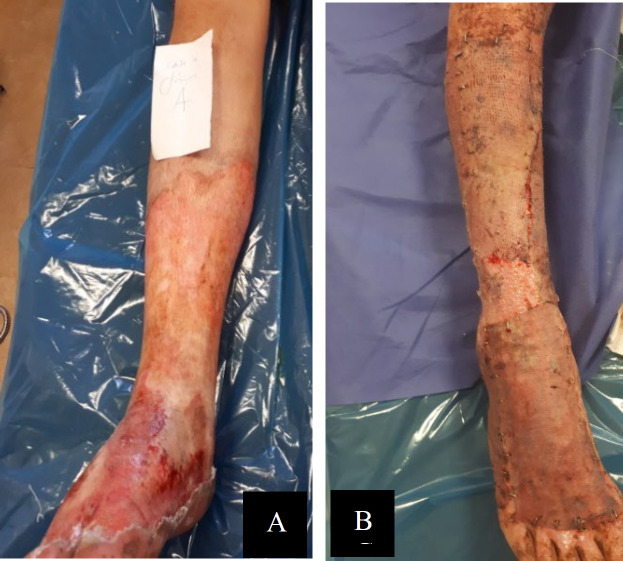
**Case 17** **:** Treatment of the right foot with *L. plantarum *supernatant: A) after 48 h and B) after 10 days of treatment as well as after skin grafting

The patient was a 30-year-old man with deep second-degree burn wounds on both feet, including the legs, ankles, and upper part of the feet. The percentage of TBSA was 18%, with dermis and epidermis involvement and skin discoloration. The patient received daily ointment containing *L. plantarum* supernatant on his right foot and silver sulfadiazine ointment on his left foot for 10 days. The patient received skin grafts on both feet. Before skin grafting, the cultures of the wound on both feet were negative. The skin graft on the right foot receiving the probiotic supernatant was successful (Figure 3). 

## Discussion

Multiple studies have concluded that probiotics are safe and could be used to treat and prevent diseases (26). Currently, one of the most important studies is related to the development of new and effective interventions for wound care. Several studies have been shown to find a new pattern for healing wounds (5, 27, 28). For example, many centuries ago, before using antiseptics and antibiotics, fermented milk was used to heal wounds (29). Over the years, many studies have found that probiotics could effectively heal wounds in humans and animals (30-32). Probiotics improve the immune system with their anti-inflammatory properties; in addition, the repairing effect of probiotics on tissues could significantly reduce wound size and inflammation (33, 34). Lactobacilli are one the most important probiotics that effectively control infection and repair burn wounds (5, 18, 19). However, there is insufficient evidence regarding the benefits of probiotics for burn patients. Therefore, this study investigated the topical treatment of second-degree burn wounds with *L. plantarum* supernatant. This study was conducted on 18 patients with burn wounds on their hands or feet. The selected patients had deep second-degree burn wounds with %TBSA between 10-20%. *L. plantarum* suspension and silver sulfadiazine ointment were used for 7 to 10 days to treat wounds in both treatment and control groups, respectively. Then the wound healing status was evaluated. The patients' wounds in the control and treatment groups were assessed in terms of the presence of bacteria. In the current study, 12 (66.7%) of wounds treated with *L. plantarum* were free from bacteria on the fifth day of treatment. Also, 14 (77.8%) of wounds treated with *L. plantarum* were free of *P. aeruginosa*. Meanwhile*, K. pneumoniae* strains were not detected in the *L. plantarum* -treated group on the fifth day of treatment. Of course, the results mentioned in this regard were not significant (Figure 1). Research has shown that probiotics have a significant inhibitory effect on MDR pathogens, especially *P. aeruginosa,* in an infected wound, which reduces the growth rate of pathogens on the skin surface, and in this way, the skin surface is less likely to be infected (7, 28). Similar studies have shown that the growth of *K. pneumoniae* and *P. aeruginosa* is dramatically reduced in the presence of cell-free supernatants of lactobacilli (18, 35). Reducing or eliminating bacteria could greatly help wound healing. In fact, the use of probiotic products induces the healing process of burn wounds in a short period of treatment due to their bactericidal effects. Therefore, these products could be important in preventing sepsis and subsequent death (5, 36).

According to the present study results, the resistance to GM was lower in *P. aeruginosa* strains isolated from the *L. plantarum* treated group than those isolated from the silver sulfadiazine treated group. Antibiotics are a prominent factor in the expansion of resistance (37). Antibiotic resistance genes (ARGs) could potentially be horizontally transferred to bacteria and contribute to the emergence of antibiotic resistance in bacteria (38). Probiotic strains do not promote the release of ARGs from their repertoire (37). In contrast, antibiotics have no conclusive effect on the number of ARGs observed. Probiotics have been shown to reduce the number of ARGs (37). It seems that the use of probiotics could reduce antibiotic resistance. However, further studies on the human gut and wound microbiota must decide on a more definitive expression.

The current study found that the need for skin grafting was similar in control and treatment groups 10 (55.6%). In this regard, Peral* et al.* (2009) showed that the need for skin grafting in patients with third-degree burns was not different between the two groups receiving *L. plantarum* and silver sulfadiazine (7). In contrast, in another study by El-Ghazely* et al.* (2016), the need for grafting was significantly different between the probiotic-treated and control groups (10% vs. 40%) (26). Perhaps the percentage and degree of burns, the microbial load of wounds, and the condition of patients were influential in this outcome.

Furthermore, in the present study, 4 (22.2%) graft rejection was observed in the control group but not in the treatment group (0%). There was a significant difference in graft rejection between the two groups (*P*=0.02) (Table 2).

Bacterial colonization and infection are the main causes of delayed wound healing and graft rejection in burns (7). Probiotics exhibit bactericidal properties by secreting antibacterial substances (39). The current study results show that the use of *L. plantarum* on wounds could help heal second-degree burn wounds and reduce graft rejection by neutralizing infections caused by *P. aeruginosa *and* K. pneumoniae*.* P.* *aeruginosa* induces higher levels of prostaglandin E2 (PGE2) compared with *L. plantarum *(7). PGE2 modulates the immune system and inflammation by regulating the expression or concentration of cytokines. It is an important mediator of inflammatory and immune responses during acute and chronic infections (40, 41). 

In addition, other studies (28, 30) have shown that lysates of tested lactic acid bacteria (LAB) stimulate keratinocyte proliferation, which accelerates re-epithelialization by inducing keratinocyte migration. In fact, probiotics enhance skin repair and healing by reducing inflammatory responses (42); in addition, graft rejection and the need for grafting are reduced using probiotics; but currently, they cannot be eliminated (7, 31).

*Lactobacillus* strains exhibit significant anti-inflammatory activity (43). It has also been reported that the acidic pH produced by *L. plantarum* cooperates with the activity of cells involved in tissue repair and immune responses (7). Of course, acidity could have harmful effects on tissues, but some compounds produced by probiotics seem to neutralize these harmful effects (7).* L. plantarum* is a microorganism that does not produce virulence factors. It easily succumbs to the host's defenses, particularly PMN (polymorphonuclear) (27). Therefore, *Lactobacillus* could be used as an important wound healing agent in the treatment of burn patients. Of course, more studies with more cases are needed to be performed in the future to achieve more effective and better treatment outcomes. The limitation of the current study is related to the number of patients. It is suggested that similar studies be conducted with a larger sample size to increase the generalizability of the results.

## Conclusion

In general, according to the results of the current study regarding the use of bacteria-free supernatant of *L. plantarum* and subsequent elimination or reduction of infection and wound healing, we could hope to achieve good results with more studies in future research in this field using topical probiotic products. If probiotic products are used as an alternative to antibiotics to reduce infection and heal wounds, it will result in higher safety, less damage, and more efficacy.

## Founding

This work was supported by a grant (No. 12925) from the Iran University of Medical Sciences.

## Conflict of Interest

The authors declared no conflict of interest.
